# Incorporating invalid test results in glass fracture strength determination

**DOI:** 10.1007/s40940-026-00331-9

**Published:** 2026-07-17

**Authors:** Mengying Peng, Andrea Franchini, Balša Jovanović, Ruben Van Coile

**Affiliations:** Ghent University, Gent, Belgium

**Keywords:** Glass fracture strength, Co-axial double ring tests, Invalid tests, Bayesian updating, Censored data, Structural reliability

## Abstract

Current standards suggest characterizing the distribution of glass fracture strength using co-axial double-ring standardized tests. While several international standards do not specify a minimum test number, ASTM C1499-19 recommends conducting at least 30 valid tests (defined as tests where fracture initiates in the inner ring). In practice, the results of invalid tests are often discarded. However, the invalid tests still hold information on the fracture strength, and neglecting them can lead to a biased estimation of the latter. This study proposes a Bayesian inference approach to extract and incorporate such information in glass strength characterization. The approach is applied to existing datasets at ambient and elevated temperatures, examining how the test number influences the coefficient of variation of the characteristic fracture strength used for design. The results show that discarding invalid tests may bias glass strength estimates. Furthermore, for the considered dataset at elevated temperatures, incorporating invalid tests enables achieving the same precision level as implied by the standard with fewer tests. These results show that the proposed approach can improve testing efficiency and refine characteristic fracture strength predictions. Finally, the study quantifies the impact of incorporating invalid tests on the structural reliability of a slab under different load ratios. Results show that ignoring invalid tests leads to an overestimation of the reliability index. This finding highlights that invalid tests should be considered for more robust reliability evaluations of glass structures.

## Introduction

The use of glass in modern buildings is increasing. In the past, this material was primarily limited to windows and other non-structural elements (Haldimann 2006), but it is now increasingly used for load-bearing applications as well (e.g., structural facades, frames) (Bedon et al. 2018; Pejatović et al. 2024). A fundamental parameter in assessing the performance, the failure behavior, and the reliability of glazing structures is the fracture strength. Because reliability is the foundation of risk assessment, accurate characterization of glass fracture strength is essential for risk-informed design and decision-making.

Much research has been conducted on glass fracture strength at ambient conditions using three-point bending (e.g., Kojima et al. 2023; Vedrtnam and Pawar 2018)) and four-point bending (e.g., Joshi and Pagni 1994; Blank et al. 1994)). Beyond these traditional test methods, the co-axial double ring (CDR) standardized test is another widely used protocol for determining glass fracture strength (EN 1288-1,2016; EN 1288-2,2016; EN 1288-5,2016; ASTM C^1499^-^19^,^2019^). Several studies have employed CDR tests for evaluating glass fracture strength at ambient temperature (e.g., Vedrtnam and Pawar 2018; Castori and Speranzini 2019; Pan et al. 2024; Naumenko et al. 2025)). Although several international CDR standards (EN 1288-1,2016; EN 1288-2,2016; EN 1288-5,2016) do not mandate a minimum number of tests, ASTM C^1499^-^19^ (2019) recommends performing at least 30 valid tests. During the CDR test, a test is only considered valid if a fracture initiates within the inner ring area. In contrast, a fracture occurring near or just outside the diameter of the inner ring is considered an invalid test (EN 1288-1,2016; EN 1288-2,2016; EN 1288-5,2016; ASTM C^1499^-^19^,^2019^), which may be caused by factors such as friction or contact stresses introduced by the load fixtures, or via misalignment of the test specimen rings (ASTM C^1499^-^19^,^2019^). In this paper, the terms “invalid points” and “valid points” are used to denote invalid and valid test results, respectively. In practice, it is commonly to exclude “invalid points” from the analysis (ISO 5275-2,1994). Next, a Weibull distribution is fit to the experimental results, which should include a minimum of 30 “valid points” (ASTM C^1499^-^19^,^2019^; ASTM C^1239^-^13^,^2024^). The 5% quantile of the obtained Weibull distribution then provides the estimate for the characteristic glass fracture strength.

The CDR testing approach described above has also been adopted for assessing glass fracture strength at elevated temperatures. However, there is still limited research on the topic. In one study, CDR tests conducted on pre-damaged soda-lime-silica glass, ranging from room temperature to 550 °C, showed that the fracture strength increases at elevated temperatures (Schwind et al. 2021). This finding has been confirmed in a more recent experimental study by Symoens et al. (2022), who conducted CDR tests at 25 °C and 275 °C. However, testing at elevated temperatures is challenging due to the glass's sensitivity to thermal shock, changing material properties, and the demanding test conditions (e.g., the time required to heat the specimen to the required temperature) (Symoens et al. 2022). In practical terms, this means that many invalid tests—ultimately excluded from analysis—are obtained as part of the test campaign, potentially leading to inefficient use of resources. Furthermore, the use of the ambient-temperature test procedure at elevated temperature lacks justification. More specifically, it is not clear whether the requirement of at least 30 valid tests is applicable.

When adopting the standard approach described above, the CDR test acts as a censoring process (Turkson et al. 2021; Meeker et al. 2022), in which the censored data (i.e., the “invalid points”) still contain incomplete or partial information (Cox 2018) on the fracture strength. Thus, while generally discarded, the “invalid points” can be exploited to enhance the accuracy of Weibull distribution and improve testing efficiency. Bayesian updating (BU) procedures provide a structured and mathematically robust approach to account for the information contained the “invalid points,” allowing for the updating of an existing probabilistic model by incorporating newly introduced data or information (Faber 2012). Thus, the scopes of this paper are to:

- Propose a Bayesian inference approach to incorporate information from “invalid points” in glass fracture strength characterization.
- Demonstrate the proposed approach through its application to existing datasets from Symoens et al. (2022).
- Investigate the effect of considering “invalid points” on test efficiency and characteristic fracture strength accuracy.
- Assess the influence of incorporating “invalid points” on the reliability evaluation of a representative glass slab.

The paper is organised as follows. Section 2 introduces the proposed Bayesian-inference-based approach, which is then applied to existing datasets in Sect. 3. Finally, conclusions are drawn in Sect. 4.

## Proposed approach

### Overview

**Fig. 1:**
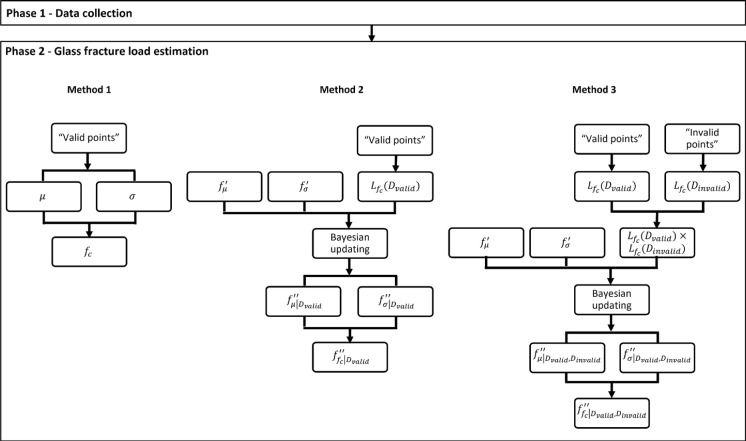
Determination of the characteristic fracture strength of a glass batch

**Fig. 2:**
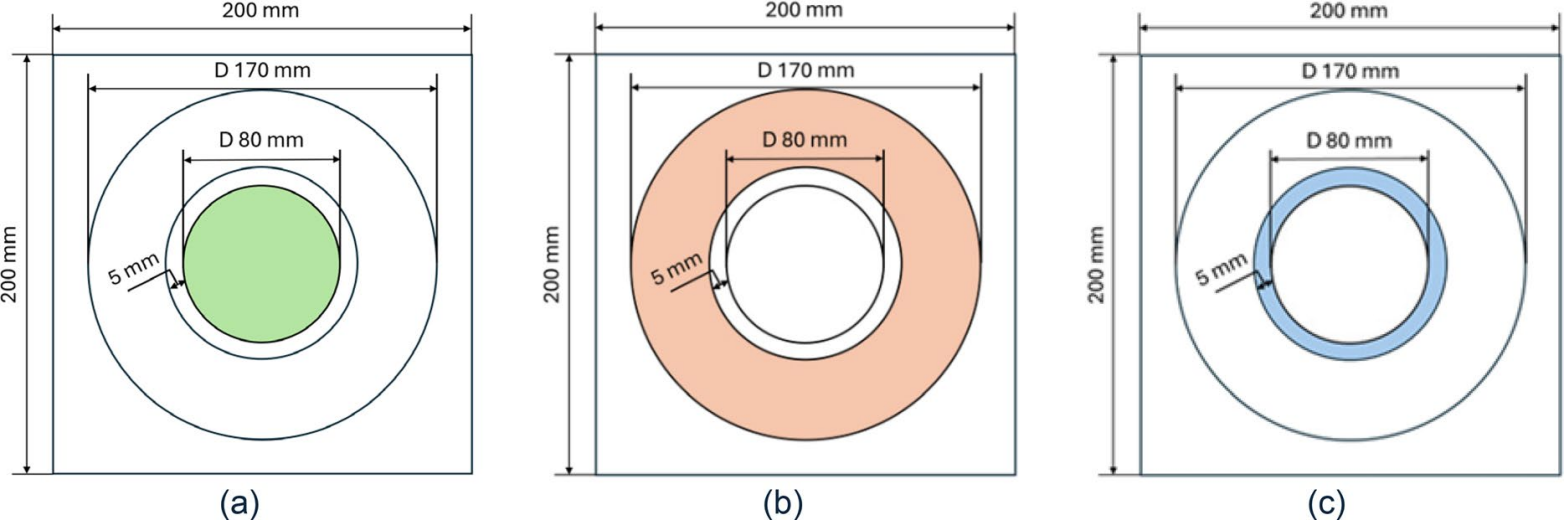
Sketches of the fracture origin locations for **a** “valid points” (fractures occurred within the inner ring); **b** excluded tests (fractures occurred outside the inner ring); **c** “invalid points” (fractures occurred below the inner ring)

The procedure to determine the characteristic fracture strength of a glass batch can be divided into two phases (see Fig. 1). Phase 1 involves data collection using the CDR testing protocol and data classification. Each test is classified as either valid or invalid based on the fracture initiation point. A hard contact may result in an invalid test result, whereby the specimen’s true strength is not reached. However, invalid data points still contain partial information about the glass fracture strength (${f}_{c}$)—specifically, that the true value exceeds the observed value. Consequently, the CDR testing can be modelled probabilistically as a censoring process (Turkson et al. 2021; Meeker et al. 2022), where observations either provide exact realizations of the true value (in the case of “valid points”; see Fig. 2a) or indicate that the true value lies within a known interval (in the case of “invalid points”). Recognizing CDR testing as a censoring process directly informs the formulation of the likelihood model in the Bayesian updating (BU) procedures introduced in Sect. 2.3.

Stronger glass samples can sustain higher loads and therefore have a higher probability to fracture before the true glass fracture strength in the inner ring is reached. Consequently, stronger samples are more likely to become “invalid points”. Excluding “invalid points” thus results in a downward shift bias in the fracture strength distribution (as not all higher strength realizations are captured in “valid points” only). The effect (conservative or unconservative) and the magnitude of this bias depend on dataset-specific characteristics, which can be accounted for using the Bayesian updating (BU) approach developed in this paper. Therefore, in general, including “invalid points” enhances the accuracy (i.e., reduces the bias) of the estimated glass fracture strength. In this paper, invalid tests with fracture initiation outside the inner ring (Fig. 2b) are excluded from the analysis, as they may be affected by spurious edge-related effects (Meyland et al. 2021), rendering them unreliable for estimating fracture strength and incompatible with the assumed censoring model. On the other hand, test results where fracture occurred below the inner ring (Fig. 2c) are considered in the proposed methodology as “invalid points,” which provide censoring information on the true fracture strength. Phase 1 concludes once 30 valid tests have been obtained.

Phase 2 uses the experimental data to estimate ${f}_{c}$. This goal can be achieved using three different methods. The first method (see Method 1 in Fig. 1), which corresponds to the standard approach, fits the experimental data to a Weibull distribution, parameterized by the sample mean and standard deviation of valid experimental results. Also including invalid results in this simple Weibull fit would be inappropriate, because those data are not observations of the true fracture strength and would bias the estimated distribution. In contrast, BU can incorporate information from invalid tests by treating the latter as censored observations. The other two methods utilize a BU procedure. Rather than relying solely on tests for estimating distribution parameters, BU allows a more refined learning process by incorporating new data to enhance existing knowledge (Gelman and Shalizi 2013). The second method (Method 2 in Fig. 1) applies BU using only “valid points.” Finally, the third method (Method 3 in Fig. 1), representing the proposed approach, employs BU to incorporate both “valid points” and “invalid points.” Further details are provided in Sects. 2.2 and 2.3.

### Standard approach (Method 1)

As recommended by ASTM standards (ASTM C^1499^-^19^,^2019^; ASTM C^1239^-^13^,^2024^), the fracture strength of glass can be modelled through a Weibull distribution with shape parameter $k$ and scale parameter $b$ (Abernethy 1998). The mean ($\mu$) and the standard deviation ($\sigma$) can be derived as follows (Abernethy 1998):

1 $$ \begin{array}{*{20}c} {\mu = b \cdot \Gamma \left( {1 + \frac{1}{k}} \right)} \\ \end{array} $$

2 $$ \begin{array}{*{20}c} {\sigma^{2} = b^{2} \left[ {\Gamma \left( {1 + \frac{2}{k}} \right) - \left( {\Gamma \left( {1 + \frac{1}{k}} \right)} \right)^{2} } \right]} \\ \end{array} $$

where $\Gamma$ is the gamma function (Abramowitz and Stegun 1948). In a traditional (frequentist) approach $\mu$ and $\sigma$ are evaluated as the sample mean and sample standard deviation of the “valid points.” The parameters $k$ and $b$ are then obtained from Eqs. (1) to (2). The standard (ASTM C^1239^-^13^,^2024^) allows to calculate the distribution parameters using either the maximum likelihood method or the method of moments. The latter method is adopted in this study. In the method of moments, the mean and variance of the assumed Weibull distribution are estimated as the mean and the variance of the test data, and the resulting equations are solved to obtain $k$ and $b$.

### Bayesian updating approaches (Method 2 and Method 3)

To enhance the predictions of ${f}_{c}$, BU can be employed. This approach incorporates information from the measured ${f}_{c}$ data in experiments ($D$) to update the prior distributions of $\mu$ and $\sigma$ using Bayes theorem (Faber 2012):

3 $$ \begin{array}{*{20}c} {f_{\mu \left| D \right.}^{^{\prime\prime}} = \frac{{L_{{f_{c} }} \left( D \right) \cdot f{\prime}_{\mu } }}{{f_{D} { }}}} \\ \end{array} $$

4 $$ \begin{array}{*{20}c} {f_{\sigma \left| D \right.}^{^{\prime\prime}} = \frac{{L_{{f_{c} }} \left( D \right) \cdot f{\prime}_{\sigma } }}{{f_{D} { }}}} \\ \end{array} $$

The posterior distributions $f_{\mu \left| D \right.}^{^{\prime\prime}}$ and $f_{\sigma \left| D \right.}^{^{\prime\prime}}$ represent the updated probability of $\mu$ and $\sigma$ after taking *D* into account. The likelihood ${L}_{{f}_{c}}(D)$ expresses how probable *D* is, assuming a Weibull distribution characterized by specific values of $\mu$ and $\sigma$. Essentially, the likelihood measures how well the Weibull distribution, given the parameters $\mu$ and $\sigma$, explains $D$ (i.e., the likelihood value will be higher if $D$ is close to the values predicted by the distribution). The prior distributions ${f}{\prime}_{\mu }$ and ${f}{\prime}_{\sigma }$ represent the initial beliefs about $\mu$ and $\sigma$, before collecting $D$. These distributions must be specified based on previous knowledge (e.g., previous experimental results, experience or professional judgment) (Faber 2012). The marginal probability ${f}_{D}$ represents the total probability of observing $D$, across all possible values of $\mu$ and $\sigma$.

The likelihood model differs for “valid points” and “invalid points.” Method 2 considers only the former type (see Fig. 1). Since “valid points” reflect the actual fracture strength of the glass material, their likelihood is described by the probability density function (PDF) of the Weibull distribution. Since the observations are independent, the total likelihood for all “valid points” is the product of the individual likelihoods for each point [see Eq. (5)].

5 $$ \begin{array}{*{20}c} {L_{{f_{c} }} (D_{valid} ) = \mathop \prod \limits_{i = 1}^{{n_{valid} }} f\left( {D_{valid,i} ;\mu , \sigma } \right) = \mathop \prod \limits_{i = 1}^{{n_{valid} }} \frac{k}{b}\left( {\frac{{D_{valid,i} }}{b}} \right)^{k - 1} exp\left( { - \left( {\frac{{D_{valid,i} }}{b}} \right)^{k} } \right)} \\ \end{array} $$

Method 3 (see Fig. 1) considers both “valid points” and “invalid points.” As defined in Sect. 2.1, “invalid points” refer specifically to test results where the fractures occurred below the inner ring, preventing the intended uniform tensile stress field from reaching its true maximum value. Therefore, in the case of “invalid points,” the glass fractures before reaching its true fracture strength inside the inner ring, so the measured value is known to be a lower bound. This behavior is characteristic of a censoring process, as mentioned in Sect. 2.1, where incomplete information is obtained due to hard contact failure. Accordingly, the likelihood of “invalid points” is expressed as the complementary cumulative distribution function (CCDF) of the Weibull distribution [see Eq. (6)], which represents the probability that the true ${f}_{c}$ exceeds the observed value. Then, assuming that each data point is observed independently of the other, the likelihood incorporates both “valid points” and “invalid points” as follows:

6 $$ \begin{gathered} L_{{f_{c} }} (D_{valid} ) \times L_{{f_{c} }} (D_{invalid} ) = \mathop \prod \limits_{i = 1}^{{n_{valid} }} f\left( {D_{valid,i} ;\mu , \sigma } \right) \times \mathop \prod \limits_{j = 1}^{{n_{invalid} }} F\left( {D_{invalid,j} ;\mu , \sigma } \right) \hfill \\ = \mathop \prod \limits_{i = 1}^{{n_{valid} }} \frac{k}{b}\left( {\frac{{D_{valid,i} }}{b}} \right)^{k - 1} exp\left( { - \left( {\frac{{D_{valid,i} }}{b}} \right)^{k} } \right) \times \mathop \prod \limits_{j = 1}^{{n_{invalid} }} exp\left( { - \left( {\frac{{D_{invalid,j} }}{b}} \right)^{k} } \right) \hfill \\ \end{gathered} $$

Computing $$f_{D} $$, which requires integrating the likelihood over all possible values of $$\mu$$ and $$\sigma$$, can be computationally expensive (Buchholz et al. 2023). Therefore, the posterior distribution is obtained by applying the Markov Chain Monte Carlo (MCMC) approach (Gelman et al. 2013), which can sample from posterior distributions that have no analytical solution without explicitly computing $$f_{D }$$(Sharma 2017).

In this study, the Random Walk Metropolis algorithm is employed (Robert and Casella 1999). The Random Walk Metropolis algorithm samples realizations from the posterior distribution by proposing new values for $\mu$ and $\sigma$ using a symmetric proposal distribution centered around the last accepted sample points. An acceptance criterion is then applied to decide whether the new values should be accepted (Robert and Casella 1999). The process is repeated until the desired number of draws is reached.

## Application to an existing dataset

### Dataset description

This section applies the approach outlined in Sect. 2 to the dataset by Symoens et al. (2022). These authors conducted CDR tests according to ASTM C^1499^-^19^ (2019) to investigate the fracture behavior and fracture strength of glass at elevated temperatures.

A total of 87 tests were performed in the experimental campaign. The specimens used in the experiments were made of annealed soda-lime-silica glass. The specimens had a width and height of 200 mm, with a nominal thickness of 4 mm. The diameters of the inner and outer rings were 80 mm and 170 mm, respectively, and the inner ring had a thickness of 10 mm. The displacement rate applied during testing was 0.05 mm/s. The CDR setup was flipped with respect to ASTM C^1499^-^19^ (2019) to ensure that the post-test fractographic examination would not be interfered with. In this flipped setup, the inner ring functions as a supporting ring while the outer ring serves as a loading ring. The CDR tests were performed at two different temperatures. Specifically, 44 tests were performed at 25 °C to obtain the experimental fracture strength distribution at ambient temperature. Then, 43 tests were performed at 275 °C to investigate the $f_{c}$ at elevated temperatures and support research into glass’s fire performance. The fracture strengths were calculated from the measured fracture loads using the linear elastic stress equation specified in ASTM C^1499^-^19^ (2019). It should be noted that the measured fracture loads of some specimens exceed the range for which the specimen geometry satisfies the small-deflection criterion of the standard. For these specimens, large-deflection effects may occur and the reported fracture strengths may therefore be overestimated. Consequently, the reported fracture strength distributions may be shifted towards higher values relative to the true distribution. This does not affect the proposed BU methodology, as the valid/invalid classification is based on the crack-initiation position rather than on the calculated fracture strength. The number of tests was driven by the code requirement to obtain 30 “valid points,” i.e., 14 and 13 “invalid points” were obtained at 25 and 275 °C. Among these “invalid points,” 6 test results were discarded at each temperature because the fractures occurred outside the expected high-stress zone where edge flaws may influence the result (see Sect. 2.1). Thus, 8 “invalid points” were retained at 25 °C and 7 at 275 °C. Further details on the datasets are provided in Table 1.

**Table 1:** Details on the experimental datasets by Symoens et al. (2022)

	25 °C	275 °C
Number of “valid points”	30	30
Number of “invalid points”	8	7
Sample mean of fracture strength (“valid points”)	92 MPa	97 MPa
Sample standard deviation of fracture strength (“valid points”)	22 MPa	39 MPa
Sample mean of fracture strength (“valid points” + “invalid points”)	92 MPa	99 MPa
Sample standard deviation of fracture strength (“valid points” + “invalid points”)	25 MPa	36 MPa

**Fig. 3:**
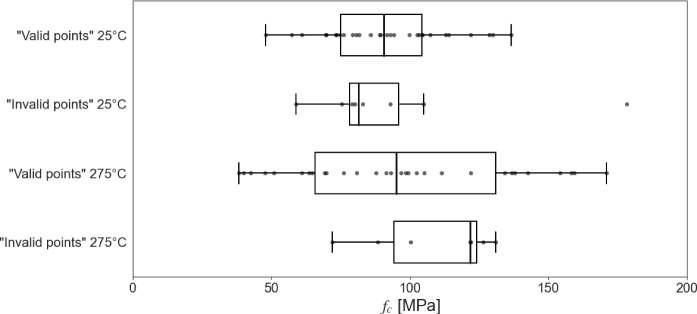
Recorded datasets from the experimental tests by Symoens et al. (2022)

Figure 3 displays the recorded data points. The fracture strength at 275 °C exhibits a larger spread, primarily observed in the interquartile range but also reflected in the whiskers, compared to the fracture strength at 25 °C. The observed median fracture strength at 275 °C is slightly higher than at 25 °C, while the maximum strength observed is markedly higher at 275 °C. When comparing “invalid points” with “valid points,” Fig. 3 primarily indicates that the “invalid points” at all temperatures distribute across a more compact range, as evidenced by the smaller interquartile range, which may indicate less variability. Moreover, “invalid points” have shorter whiskers, indicating also a more restricted spread compared with “valid points.”

**Fig. 4:**
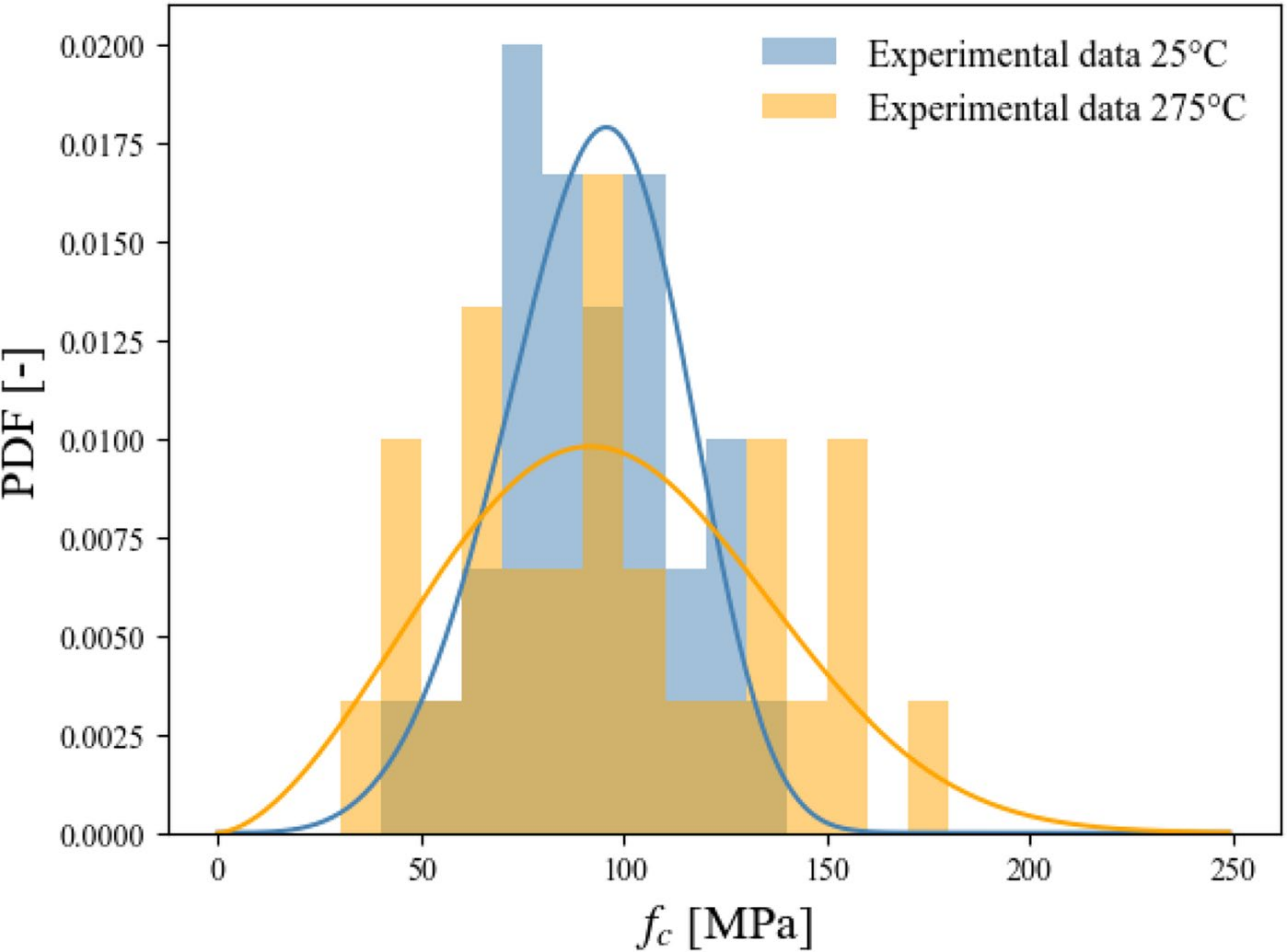
Fracture strength data from Symoens et al. (2022) (25 and 275 °C) and fitted Weibull distributions

In the standard approach (Method 1 in Fig. 1), the fracture strength is modeled using a Weibull distribution, whose parameters are estimated from valid data points via the method of moments. The results are shown in Fig. 4.

### Bayesian updating

In the BU approaches (Method 2 and 3 in Fig. 1), the $\mu$ and $\sigma$ are treated as uncertain parameters which are to be updated considering the available experimental data. The prior distribution represents the initial knowledge about the parameters before considering the experimental data and is, by definition, independent of the experimental data used for BU. The prior distributions of $\mu$ and $\sigma$ are considered to be uniform and with wide bounds, reflecting that little information about their true values is known upfront. A sensitivity analysis confirmed that the chosen prior bounds are sufficiently wide so that they do not influence the posterior distributions. Conversely, if the priors were too narrow, the posterior distributions could be biased by excluding parameter values that the experimental data may support. More precisely, for $\mu$, the uniform distribution is defined within the range [40, 200 MPa]. For $\sigma$, the prior uniform distribution is specified by the range [10, 100 MPa]. The selection of these ranges for the prior will not affect the BU results as long as the bounds are sufficiently wide relative to the experimental data and sufficient data becomes available (Gelman et al. 2013). An overview of the calculation input for BU procedure is given in Table 2. The prior distributions of $\mu$ and $\sigma$ are considered to be independent. The number of draws is determined based on the convergence of multiple chains: three separate Markov chains are generated with different starting points to check the convergence under the selected number of draws, which is 12^4^. In this study, the first 10,368 (50%) of the samples are discarded as the “burn-in” period, resulting in 10,368 samples. The jump sizes within the RWM algorithm for both $\mu$ and $\sigma$ are set to 8 MPa at 25 °C, while at 275 °C, they are set to 10 MPa. The resulting acceptance rates are between 0.2 and 0.4 (see specific acceptance rate results in Table 3).

**Table 2:** Model input for Bayesian updating

	25 °C	275 °C
Prior distributions	$\mu$ ~ U (40, 200 MPa)	$\mu$ ~ U (40, 200 MPa)
	$\sigma$ ~ U (10, 100 MPa)	$\sigma$ ~ U (10, 100 MPa)
Likelihood	Weibull	Weibull
Starting point	$\mu$: 90 MPa; $\sigma$: 20 MPa	$\mu$: 90 MPa; $\sigma$: 20 MPa
Number of draws	12^4^	12^4^

**Table 3:** Acceptance rates

	25 °C	275 °C
“Valid points”	0.23	0.34
“Valid points” + “invalid points”	0.31	0.37

**Fig. 5:**
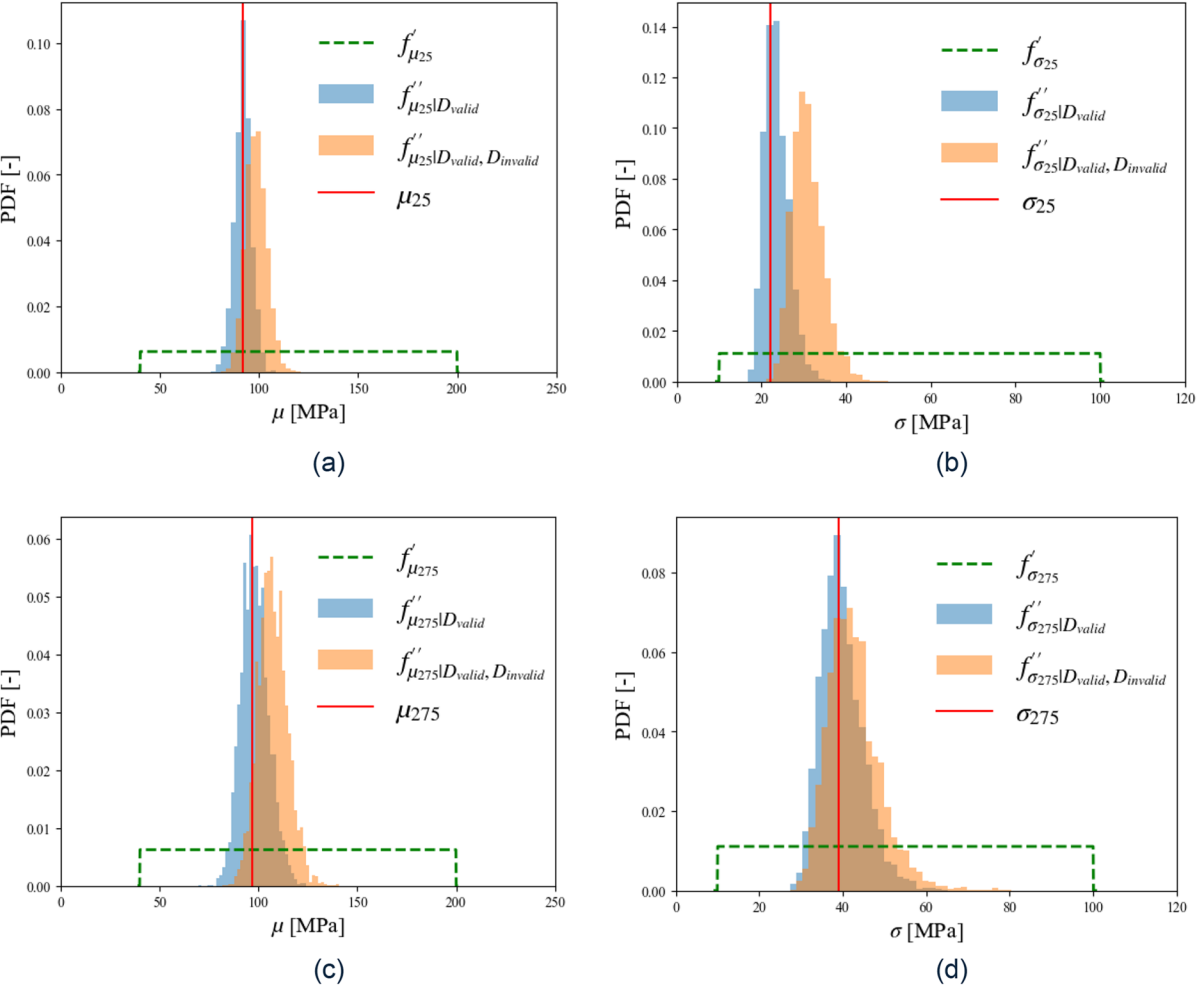
Prior and posterior distributions of fracture strength mean and standard deviation: **a**, **b** 25 °C; **b**, **c** 275 °C

**Fig. 6:**
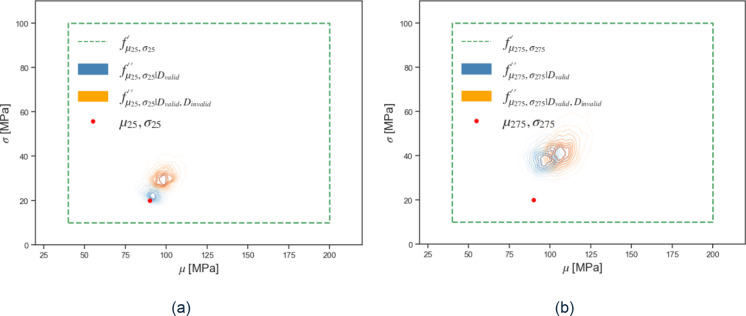
The joint prior and posterior distributions of fracture strength mean and standard deviation: **a** 25 °C; **b** 275 °C

Figure 5 presents the prior ($f{\prime}_{\mu ,25}$, $f{\prime}_{\sigma ,25}$, $f{\prime}_{\mu ,275}$, $f{\prime}_{\sigma ,275}$) and posterior distributions of fracture strength mean and standard deviation for experimental campaigns at 25 and 275 °C. The posterior distributions are derived by updating with “valid points” only (Method 2 in Sect. 2.3; see $f_{{\mu ,25\left| {D_{valid} } \right.}}^{^{\prime\prime}}$, $f_{{\sigma ,25\left| {D_{valid} } \right.}}^{^{\prime\prime}}$, $f_{{\mu ,275\left| {D_{valid} } \right.}}^{^{\prime\prime}}$,$f_{{\sigma ,275\left| {D_{valid} } \right.}}^{^{\prime\prime}}$) as well as with both “valid points” and “invalid points” (Method 3 in Sect. 2.3; see $f_{{\mu ,25\left| {D_{valid} } \right., D_{invalid} }}^{^{\prime\prime}}$, $f_{{\sigma ,25\left| {D_{valid} } \right., D_{invalid} }}^{^{\prime\prime}}$, $f_{{\mu ,275\left| {D_{valid} } \right., D_{invalid} }}^{^{\prime\prime}}$, $f_{{\sigma ,275\left| {D_{valid} } \right., D_{invalid} }}^{^{\prime\prime}}$). The reference lines show the sample mean ($\mu_{25}$, $\mu_{275}$) and sample standard deviation ($\sigma_{25}$, $\sigma_{275}$) of the Weibull distribution obtained by Symoens et al (2022) considering the procedure in ASTM C^1499^-^19^ (2019) (see Method 1 in Fig. 1 and distributions in Fig. 4). The joint prior and posterior distributions of $\mu$ and $\sigma$ are visualized in Fig. 6.

The posterior distributions based solely on “valid points” are concentrated around the reference values. This concentration is the expected outcome when using “valid points” since the same data was used in the frequentist approach of Symoens et al. (2022). Considering both “valid points” and “invalid points,” however, shifts the distributions of the mean and standard deviation to larger values. A larger $$\mu$$ indicates a higher expected value of $$f_{c}$$. Larger $$\sigma$$ indicates more variability. The rightward shift suggests a bias in the standard requirements. Specifically, the results of Figs. 5 and 6 indicate that the procedure whereby “invalid points” are removed from the dataset predominantly omits stronger samples from the dataset. The occurrence of “invalid points” could, for example, relate to the failure of the rubber rings used in the CDR at high load levels to avoid hard contact, as described in the test setup in Symoens et al. (2022), thus creating a bias in favour of a weaker assessment of fracture strength. This bias cannot be dismissed as conservative, since the results indicate that the omission of “invalid points” also leads to an underestimation of the fracture strength variability (i.e., standard deviation). At 275 °C, however, the shift of $$\sigma$$ for the experimental campaign is small, indicating the uncertainty in $$f_{c} $$ is not significantly affected by the consideration of both “valid points” and “invalid points.”

Figure 6 furthermore shows that the contours are approximately circular for both ambient and elevated temperatures, suggesting no major linear correlation between $\mu$ and $\sigma$ after BU. This is supported by Pearson correlation coefficients (Pearson 1895) of 0.210 and 0.344 at 25 and 275 °C, respectively. However, mutual information values (Shannon 1948) of 4.736 and 4.093 for 25 and 275 °C, respectively, suggest a strong non-linear dependence between the posterior distributions of $\mu$ and $\sigma$. The non-linear dependency between $\mu$ and $\sigma$ however needs to be considered in pre-posterior Bayesian analysis and sequential Bayesian experimental design frameworks (Rainforth et al. 2024). However, in the proposed approach, BU is performed only once for a given set of data points. Therefore, neglecting the correlation between the two parameters is acceptable in this paper.

**Fig. 7:**
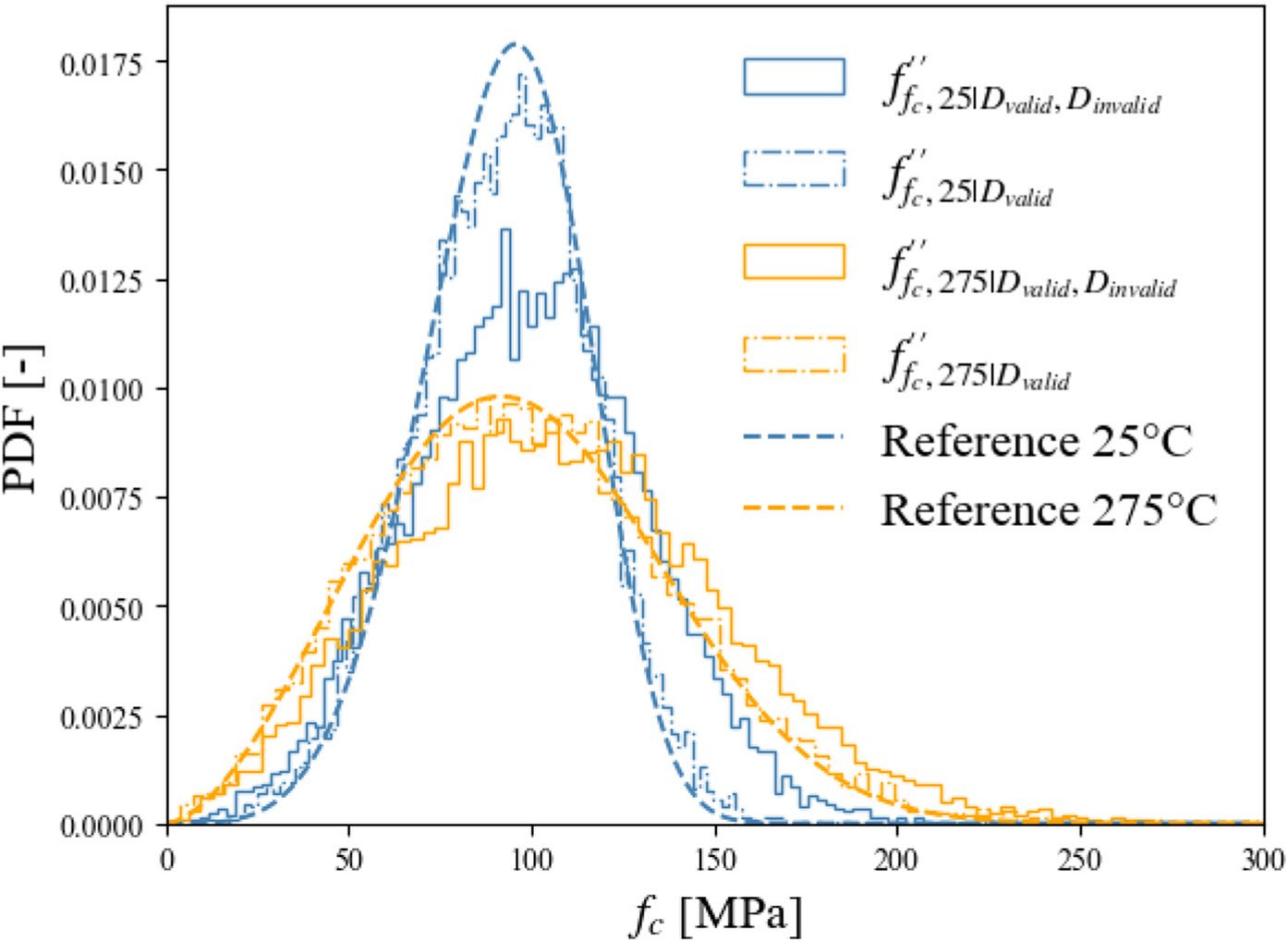
Updated glass fracture strength ($f_{c}$) distributions (PDF), compared with the references for experimental campaigns at 25 and 275 °C

**Fig. 8:**
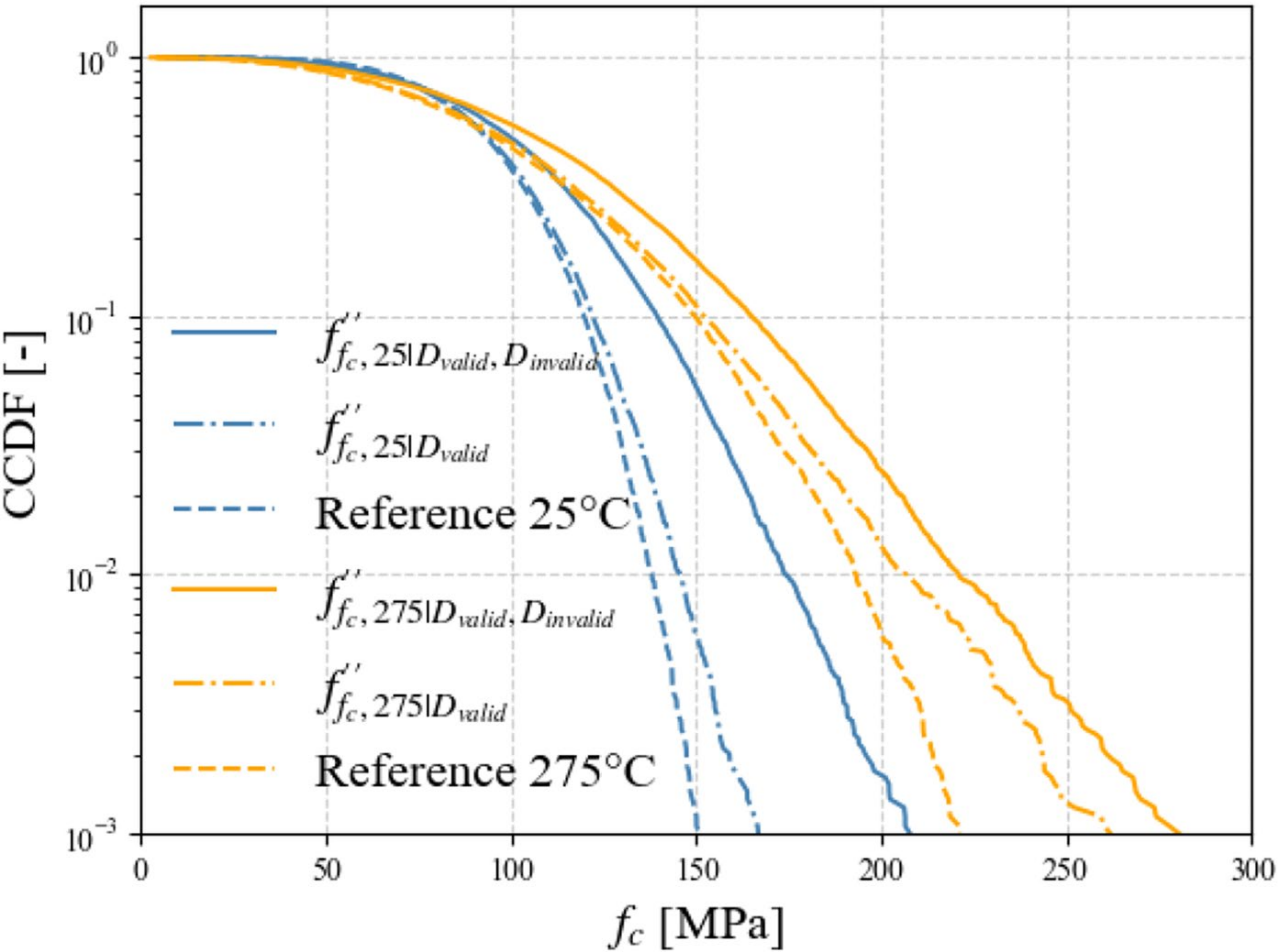
Updated glass fracture strength ($f_{c}$) distributions (CCDF), compared with the references for experimental campaigns at 25 and 275 °C

**Fig. 9:**
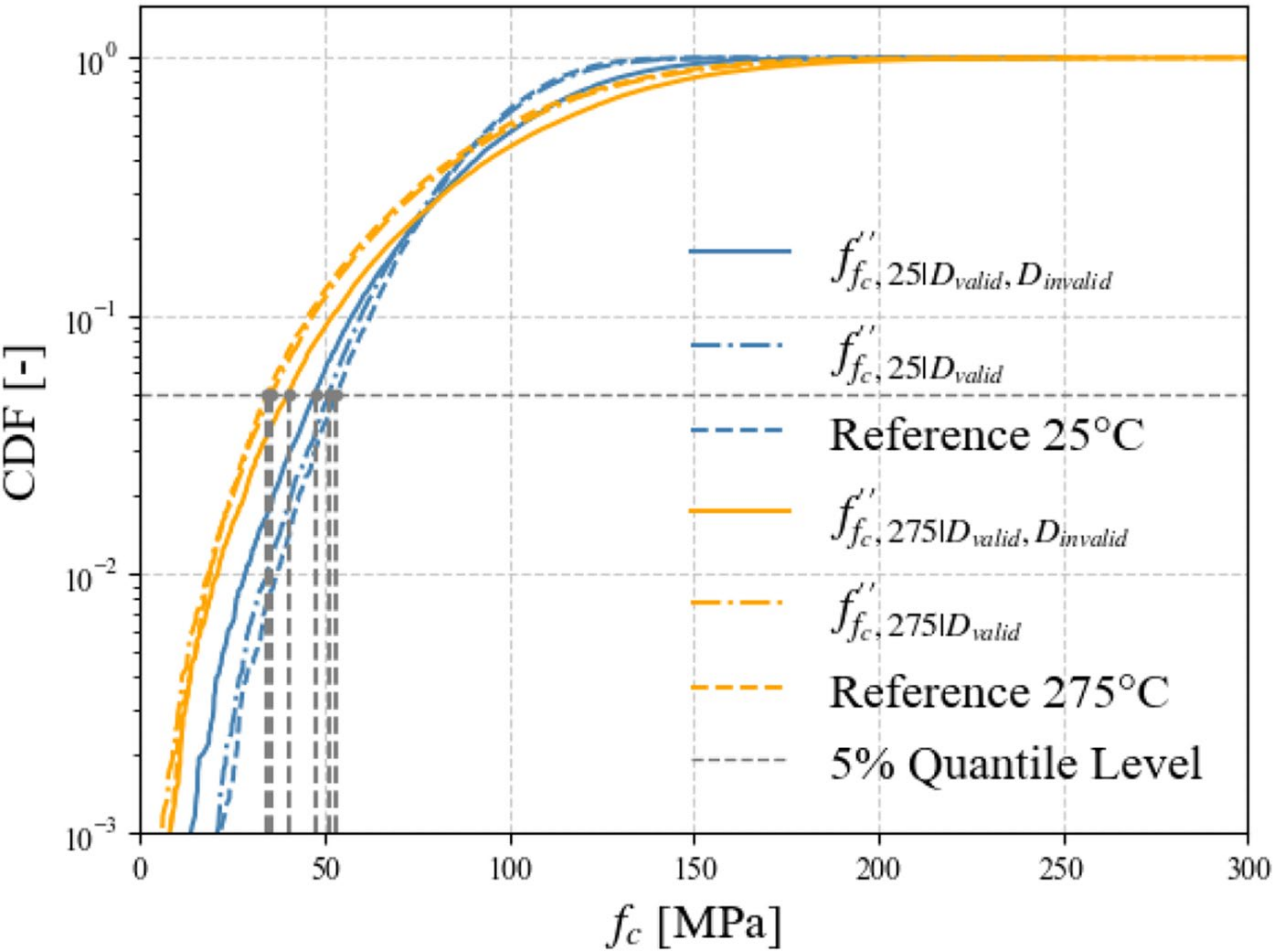
Updated glass fracture strength ($f_{c}$) distributions (CDF), including their 5% quantiles, compared with the references for experimental campaigns at 25 and 275 °C

The posterior predictive distributions of $$f_{c}$$, obtained by applying inverse CDF to the posterior sample pairs of $$\mu$$ and $$\sigma$$, are compared with the reference distributions (the fitted Weibull distributions based on the experimental data) in Figs. 7, 8 and 9. The $$\mu$$ and $$\sigma$$ of the posterior $$f_{c} $$ distributions are displayed in Table 4. The wider distributions in Fig. 7 and larger $$\sigma$$ in Table 4 indicate that incorporating “invalid points” in the BU procedure (Method 3) increases the spread of the distributions. This increase is more clearly visualized in Fig. 8 by observing the width of the upper tail. The higher variability observed in the Bayesian evaluation, which considers both “valid points” and “invalid points” (Method 3), again indicates a bias in the test procedure where “invalid points” are excluded from the analysis. However, from Fig. 9, at 25 °C the 5% quantile (usually taken as the characteristic strength) decreases significantly when “invalid points” are also considered (Method 3), dropping from 51 to 47 MPa—an 8% reduction. This indicates that the actual characteristic strength is lower than the one obtained following the standard (53 MPa; Method 1). On the other hand, for the experimental campaign at 275 °C, the 5% quantile increases from 35 to 40 MPa—a 13% rise—when incorporating “invalid points” (Method 3). In this case, considering only “valid points” (Method 2) or using the standard value (35 MPa; Method 1) underestimates the characteristic strength.

**Table 4:** Mean and standard deviation of posterior glass fracture strength (${\mathrm{f}}_{{\mathrm{c}}}$) distributions for experimental campaigns at 25 °C and 275 °C

	$\mu$ (MPa)	$\sigma$ (MPa)
Reference 25 °C	92	22
$f_{{f_{c,25} \left| {D_{valid} } \right.}}^{^{\prime\prime}}$	92	24
$f_{{f_{c,25} \left| {D_{valid} ,D_{invalid} } \right.}}^{^{\prime\prime}}$	99	32
Reference 275 °C	97	39
$f_{{f_{c,275} \left| {D_{valid} } \right.}}^{^{\prime\prime}}$	99	41
$f_{{f_{c,275} \left| {D_{valid} ,D_{invalid} } \right.}}^{^{\prime\prime}}$	108	44

### Rationalizing the number of tests

The ASTM C^1499^-^19^ (2019) recommends a minimum of 30 valid tests to estimate the Weibull distribution parameters. This section investigates whether incorporating “invalid points” allows (1) achieving the same precision level as implicitly required by the standard with fewer tests and (2) attaining a higher precision level (i.e., narrower confidence interval) with the same number of tests required by the standard. The goal of this section is to illustrate the potential effect and magnitude, both of which are dataset-specific, of the bias introduced by ignoring “invalid points.” As a result, the specific numerical outcomes should not be generalized to other test series. Furthermore, the section demonstrates that the BU-based methods can allow for more efficient testing, in addition to reducing the bias introduced when only considering valid tests.

While ASTM C^1499^-^19^ (2019) recommends a minimum of 30 valid tests at ambient temperature, it does not explicitly state a required precision level. In this paper, the term “precision level” refers to the coefficient of variation (CoV) of the 5% quantile ($$f_{c,5\% }$$) of the $$f_{c} $$ distribution. The precision level associated with the 30 valid tests is thus implicit, and referred to as the “precision level implicitly entailed by the standard” in the remainder of the paper. Here, it must be recognized that the reference to a precision level implicitly required in the standard testing approach is ultimately misleading, as it quantifies certainty (i.e., statistically confidence) around an incorrect estimate due to the bias induced by discarding “invalid points.” However, despite its bias, this precision level provides a benchmark for evaluating whether the test sequences—after incorporating “invalid points” (Method 3)—still meet the implicit standard requirement.

$f_{c,5\% }$ is generally used as the characteristic value for code-based design (EN 572-1,2016), indicating that there is a 95% probability that the actual glass strength will be greater than or equal to the specified strength. However, $f_{c,5\% }$ itself is not deterministic: it is a statistical estimate derived from finite test data and thus carries its own uncertainty. In the Bayesian approaches (Method 2 and 3), this uncertainty is explicitly accounted for by assigning probability distributions to the distribution parameters (i.e., $\mu$ and $\sigma$) rather than treating them as fixed values. Therefore, the characteristic glass strength can also be represented through a distribution. As part of the MCMC sampling for BU, pairs ($\mu_{i}$*,* $\sigma_{i}$) of values sampled from the posterior distribution are obtained. Each pair defines a different Weibull distribution and, therefore, a different $f_{c,5\% }$. In the following, the obtained precision level of $f_{c,5\% }$ is quantified in terms of its coefficient of variation, $CoV\left( {f_{c,5\% } } \right)$.

The following steps are followed to investigate the effect of the number of tests on $CoV\left( {f_{c,5\% } } \right)$:

- At each temperature (25 and 275 °C), 100 random sequences of observed data are generated from the available datasets. Each sequence is a reshuffled version of the test results obtained in Symoens et al. (2022) and contains both valid and invalid data points.
- For each sequence, the prior distributions of $\mu$ and $\sigma$ are sequentially updated using the observed data, starting with the first data point and progressively incorporating additional points one at a time until the entire sequence has been utilized.
- Each update provides posterior samples of the mean and standard deviation. For each sample pair *k*, $f_{c,5\% ,k}$ is obtained. The mean and standard deviation of $f_{c,5\% }$ are obtained considering all posterior samples. Then, the $CoV\left( {f_{c,5\% } } \right)$ is calculated by dividing the sample standard deviation by the sample mean.
- This process is conducted twice: first by updating the distributions using only “valid points” (Method 2 in Sect. 2.3), and then by incorporating both “valid points” and “invalid points” (Method 3 in Sect. 2.3).

The described approach provides insights into how the order in which the data are observed affects the variability of $f_{c,5\% }$. The occurrence of valid and invalid points is a random process. By considering alternative ordering for the samples, the described approach provides insights into how the order in which the data are observed affects the variability of $f_{c,5\% }$. In particular, different sequences lead to different intermediate estimates of $f_{c,5\% }$: a series of points close to each other tends to shrink the posterior distribution, whereas points that are more widely separated lead to a widening of the posterior distribution. However, as more data are incorporated, the influence of any single observation diminishes, and the updated values converge to the same result once all data have been included. However, it is noted that this approach only reshuffles existing datasets. Repeating the test series would produce different test values and could therefore lead to different outcomes.

**Fig. 10:**
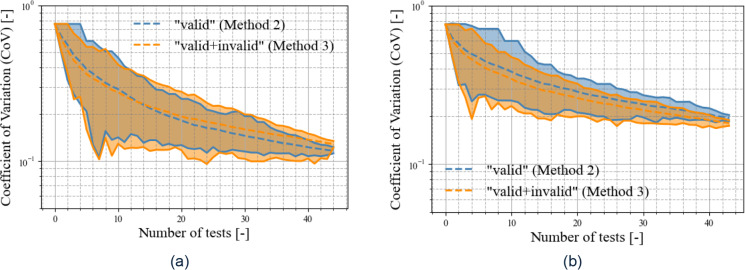
“Coefficient of Variation (CoV) of 5% quantile vs Number of tests” in two strategies, showing a cloud of 100 sequences and the associated mean value at 25 °C (**a**) and 275 °C (**b**)

Figure 10 shows the results for the generated random sequences at the two testing temperatures. The clouds represent envelopes of the $CoV\left( {f_{c,5\% } } \right)$ results from the 100 sequences. The dashed lines show the mean $CoV\left( {f_{c,5\% } } \right)$ across the 100 randomized sequences. The starting point of the $CoV\left( {f_{c,5\% } } \right)$ at zero tests is calculated based on 10^5^ samples from the prior distributions, resulting in a CoV value of 0.76. For a given temperature and updating rule (“valid” only, i.e., Method 2, or “valid + invalid,” i.e., Method 3), all sequences converge to the same $CoV\left( {f_{c,5\% } } \right)$ after considering all the test data. This is because, at that point, the information contained in each sequence is the same (i.e., all the available information in the dataset is considered). The $CoV\left( {f_{c,5\% } } \right)$ envelopes at 275 °C (Fig. 10b) remain higher those at 25 °C in Fig. 10a, indicating that the higher test temperature leads to greater uncertainty in $f_{c,5\% }$.

By comparing the orange and blue mean curves in Fig. 10a, it is observed that at 25 °C, considering both “valid points” and “invalid points” (Method 3) does not markedly influence the average uncertainty in the characteristic fracture strength during the early testing stage. However, the average uncertainty increases in the later testing stage compared with considering only “valid points” (Method 2). Therefore, for experimental campaign at 25 °C, considering “invalid points” does not give an advantage in reducing test numbers, but in reducing bias (see Sect. 3.2). On the other hand, Fig. 10b shows that, at 275 °C, the $$CoV\left( {f_{c,5\% } } \right)$$ obtained by considering both “valid points” and “invalid points” (Method 3) is typically lower than that derived from using only “valid points” (Method 2). This observation suggests that incorporating information from “invalid points” can reduce uncertainty on $$f_{c,5\% }$$ at the elevated temperature. It is noteworthy that the reduction in $$CoV\left( {f_{c,5\% } } \right)$$ occurs despite the fact that the $$f_{c} $$ distribution becomes wider when incorporating the “invalid points,” as previously described in Figs. 7 and 8. Due to the constantly higher precision level at different numbers of tests, incorporating “invalid points” at 275 °C allows for achieving a smaller CoV with the same total number of tests. If the bias caused by excluding “invalid points” is ignored, the improved precision level in $$f_{c,5\% } $$ can be interpreted as an indicator of uncertainty reduction, which reflects in an average decrease from 0.321 to 0.295 at 275 °C in the $$CoV\left( {f_{c,5\% } } \right) $$ across 100 sequences over the total number of tests. Consequently, the same precision level as entailed by the 30-valid-test requirement can be obtained with fewer tests by incorporating the information carried by “invalid points.”

When updating based only on “valid points” (Method 2), $CoV\left( {f_{c,5\% } ,30 \, valid} \right)$ represents the precision level implicitly entailed by the standard. In Fig. 10, $CoV\left( {f_{c,5\% } ,30 \, valid} \right)$ is a single value at a given temperature, as it contains all valid information available in the dataset. This is the single CoV value obtained when considering all the “valid points.”

**Fig. 11:**
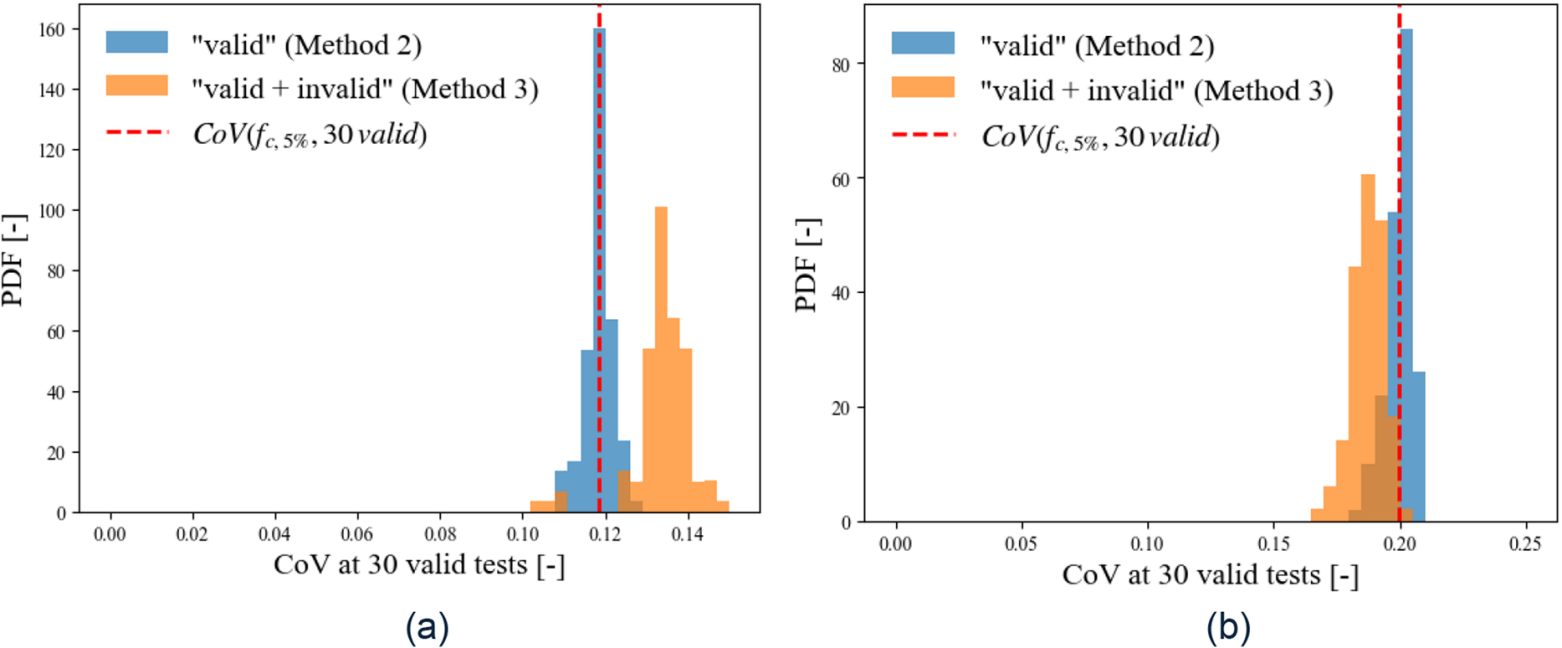
Distributions of Coefficient of Variation (CoV) at 30 valid tests when considering only “valid points” (Method 2) and incorporating “invalid points” (Method 3) at 25 °C (**a**) and 275 °C (**b**), compared with implicit standard requirement

Figure 11 illustrates the distributions of CoV at 30 valid tests when employing Method 2 and Method 3. The rightward shift in the distribution after considering “invalid points” (Method 3) in Fig. 11a highlights a bias at ambient temperature caused by the underestimation of uncertainty when excluding “invalid points” (Method 2). Figure 11b shows that $CoV\left( {f_{c,5\% } ,30 \, valid} \right)$ can generally be achieved with fewer tests when “invalid points” are included, suggesting enhanced test efficiency at the elevated temperature (275 °C).

From Figs. 10 and 11, additionally, the approach refines the characteristic fracture strength predictions under the elevated temperature (275 °C) by achieving a smaller CoV with the same number of tests as required by the code for experimental campaign at 275 °C.

The same precision level as implicitly required by the standard is considered to be achieved in test sequences where “invalid points” are also taken into account at the point where $CoV\left( {f_{c,5\% } ,valid + invalid} \right) = CoV\left( {f_{c,5\% } ,30\, valid} \right)$. The values of $CoV\left( {f_{c,5\% } ,30 \, valid} \right)$ are 0.1186 at 25 °C and 0.1997 at 275 °C. The associated total number of tests meeting this criterion is denoted as $N_{total,CoV}$_._ The number of “valid points” within this sample is denoted as $N_{valid,CoV}$. Across 100 sequences, when considering only “valid points” (Method 2), some of the sequences do not achieve the implicit standard requirement. At 25 °C, this also occurs when applying Method 3, which incorporates invalid tests as part of the updating procedure. However, at 275 °C, the precision level obtained when considering only 30 valid tests is already achieved when applying Method 3 at an average of 25 valid tests and a total of 36 tests. The result demonstrates that at 275 °C, when incorporating “invalid points,” the same precision level as implicitly required by the standard can be achieved with fewer tests.

**Fig. 12:**
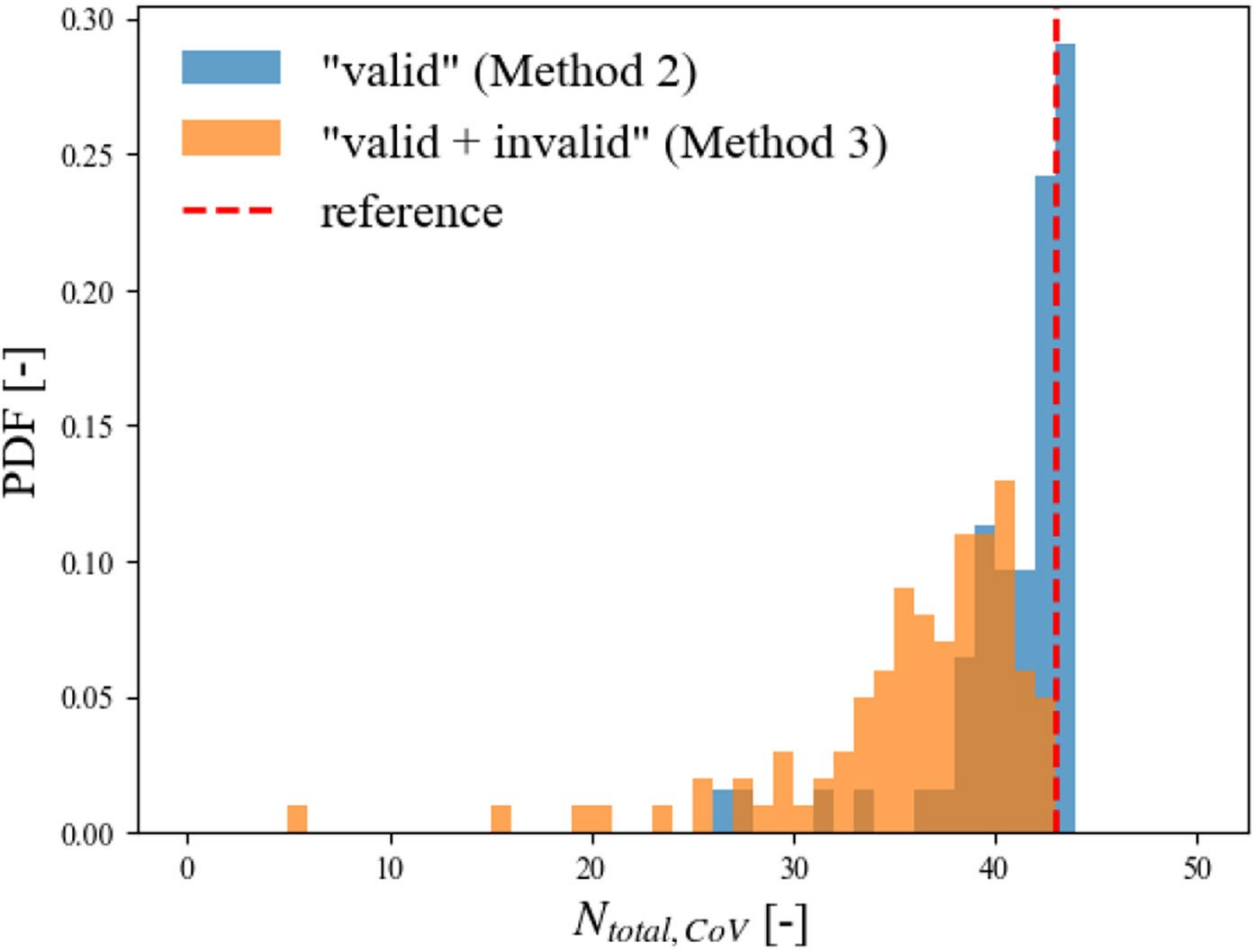
Distributions of total number of tests achieving implicit standard requirement ($N_{total,CoV}$) when applying Method 2 and Method 3, compared with the reference average (275 °C)*. *Note: When considering only “valid points” (Method 2), some of the sequences do not achieve the target precision level

At 275 °C, each of the 100 sequences requires a different number of total tests ($N_{total,CoV}$) to obtain the same precision level as implicitly required by the standard, i.e., $CoV\left( {f_{c,5\% } ,30 \, valid} \right)$, via Method 2 and Method 3. Figure 12 shows the distributions of $N_{total,CoV}$ when applying Method 2 and Method 3 at 275 °C. However, some of the sequences do not achieve $CoV\left( {f_{c,5\% } ,30 \, valid} \right)$ when employing Method 2. Figure 12 shows that the distribution incorporating “invalid points” (Method 3) is shifted to the left relative to the distribution using only “valid points” (Method 2). This left shift indicates that at 275 °C, in expectation, fewer tests are needed to achieve $CoV\left( {f_{c,5\% } ,30 \, valid} \right)$ after considering “invalid points.” These results demonstrate that the proposed Bayesian inference approach can improve the test efficiency for the experimental campaign at 275 °C. Given the observations from the experimental campaign at 25 °C in Fig. 10a, where the average $CoV\left( {f_{c,5\% } } \right)$ increases in the later testing stage after incorporating “invalid points” (Method 3), the improvement in test efficiency appears to become more significant at higher test temperatures.

In conclusion, BU offers clear advantages in refining predictions and improving test efficiency, making it a valuable tool for assessments of $f_{c}$. For fracture strength evaluations, the increased efficiency was observed for the elevated-temperature dataset. At ambient temperature, considering “invalid points” is crucial for reducing bias in fracture strength assessment.

### Reliability assessment of a glass slab

This subsection uses the glass fracture strength distributions obtained in Sect. 3.2 to assess the influence of incorporating “invalid points” on structural reliability.

The reliability calculation approach adopted in this study is based on the stresses in the first (tensioned) glass layer of a simply supported slab panel in bending, considering different assumed load ratios. Instability effects are considered not to govern the glass failure. Considering the above, the results apply equally to a single glass pane or to multi-layered laminated glass and no assumptions need to be made regarding the regarding full or non-composite interaction between glass plies or time-dependent interlayer behaviour. The only necessary assumption is that the first glass layer has a thickness of 4 mm, to remain consistent with the available experimental strength data by Symoens et al (2022). This is because the CDR-derived fracture strength is thickness-dependent (Castori and Speranzini 2019).

The structural reliability is quantified by the reliability index $\beta$, see Eq. (7) (Holicky 2009), which is derived from the probability of failure ($P_{f}$), shown in Eq. (8) (Holicky 2009):

7 $$ \begin{array}{*{20}c} {\beta = - \phi^{ - 1} \left( {P_{f} } \right)} \\ \end{array} $$

8 $$ \begin{array}{*{20}c} {P_{f} = P\left( {K_{E} \cdot E > K_{R} \cdot R} \right)} \\ \end{array} $$

where $$\phi^{ - 1}$$ is the inverse standard normal distribution; $$E$$ is the load effect, which is here the stress $$\sigma_{E}$$ in the first glass layer; $$R$$ is the simulated resistance, which is here the glass fracture strength $$\sigma_{g}$$; $$K_{R}$$ and $$ K_{E}$$ are model uncertainty factors for the resistance and the load effect, respectively.

Assuming 100% design utilization (i.e., the design load effect $\sigma_{E,d}$ is equal to the design resistance $\sigma_{g,d}$) and for a given load ratio $\chi = \frac{{\sigma_{Q,k} }}{{\sigma_{G,k} + \sigma_{Q,k} }}$, the characteristic stresses from permanent load $\sigma_{G,k}$ and live load $\sigma_{Q,k}$ can be obtained from the material strength parameters using Eq. (9). This equation follows the structural form of the design equation in EN 16612 and is applied in combination with the action combinations given in EN 1990 (2002) [Eq. (10)].

9 $$ \begin{array}{*{20}c} { \sigma_{g,d} = \frac{{\eta \cdot \sigma_{g,k} }}{{\gamma_{M;A} }}} \\ \end{array} $$

In Eq. (9), $\sigma_{g,k}$ is the characteristic glass bending strength [MPa] and corresponds to the 5% quantile of the fracture strength distribution ($f_{c,5\% }$) obtained in Sect. 3.2; $\eta$ is a factor accounting for size, stress field, loading rate, and surface conditions effects. For the scope of this example (i.e., investigating the effect of excluding “invalid points” on reliability assessment), three values of $\eta$ are assumed for comparison (i.e., 0.25, 0.45, and 0.65); $\gamma_{M;A}$ is the material partial factor from EN 16612 (2019), which equals 1.8 for annealed glass.

10 $$ \begin{array}{*{20}c} {\sigma_{E,d} = max\left\{ {\left( {\gamma_{G} \sigma_{G,k} + \gamma_{Q} \psi \sigma_{Q,k} } \right);\left( {r_{d} \gamma_{G} \sigma_{G,k} + \gamma_{Q} \sigma_{Q,k} } \right)} \right\}} \\ \end{array} $$

In Eq. (10), $\gamma_{G}$ is the partial factor for permanent load, which is 1.35; $\sigma_{G,k}$ is the characteristic stress from permanent load [MPa]; $\gamma_{Q}$ is the partial factor for live load, which equals 1.5; $\psi$ is the combination factor for accompanying variable load, which is taken as 0.7 (value for residential and office areas); $\sigma_{Q,k}$ is the characteristic stress from live load [MPa]; $r_{d}$ is the reduction factor for favourable permanent actions, and is equal to 0.85. The distributions of the load effect is finally obtained by (Holicky 2009):

11 $$ \begin{array}{*{20}c} {\sigma_{E} = \sigma_{G} + \sigma_{Q} } \\ \end{array} $$

The probabilistic models for the model uncertainties and the load variables are shown in Table 5, based on Jovanović et al. (2021) and Holicky (2009).

**Table 5:** Probabilistic models for the model uncertainties and the load variables for reliability analysis

	Description	Distribution	μ	CoV	Units
$\sigma_{G}$	Permanent load stress	Normal	$\sigma_{G,k}$	0.1	[MPa]
$\sigma_{Q}$	Live load stress	Gumbel	$0.6 \cdot \sigma_{Q,k}$	0.35	[MPa]
$K_{R}$	Resistance uncertainty	Lognormal	1.1	0.1	[–]
$$ K_{E}$$	Load effect uncertainty	Lognormal	1.0	0.1	[–]

**Fig. 13:**
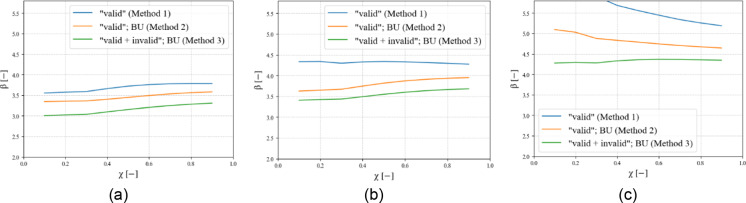
Reliability index ($\beta$) as a function of load ratios ($\chi$) for different values of $\eta$: **a** $\eta = 0.65$, **b** $\eta = 0.45$, and **c** $\eta = 0.25$

Figure 13 shows the variation of $\beta$ with $\chi$ at ambient temperature when applying the fitted Weibull distribution (Method 1), when considering only “valid points” (Method 2), and when considering both “valid points” and “invalid points” (Method 3). It can be seen that, when applying the standard method (Method 1), a reliability index higher than that obtained from Method 2 and Method 3 is observed. This is because the fracture strength distributions obtained from the BU approaches (see Fig. 7 in Sect. 3.2) exhibit a heavier lower tail (i.e., contains more weak samples). This leads to higher $P_{f}$ and thus lower $\beta$. Similarily, Method 2 (using BU but ignoring “invalid points”) leads to an overestimation of $\beta$ across the full range of $\chi$ values. This overestimation indicates an unsafe evaluation in this case study.

Based on these results, excluding “invalid points” (Methods 1 and 2) can introduce an unsafe bias in reliability analysis. The impact on reliability depends on the effect on both the mean and standard deviation of the glass strength, which is dependent on the specific samples in the test series. While the mean value is always underestimated when neglecting “invalid points” (see discussion on bias in Sect. 2.1), the probability of low strength realizations can still be higher because the variability of the fracture strength distribution can increase after considering “invalid points” (Method 3). By incorporating “invalid points” (Method 3), the assessment more accurately reflects the actual performance of the glass slabs under varying load ratios as it accounts for the censored observations, thereby reducing the bias.

Overall, this section shows that “invalid points” should be considered in glass fracture strength characterization to avoid misleading safety evaluations and ensure unbiased input for reliability assessment.

## Conclusions

The standard CDR testing approach for experimentally determining glass fracture strength distinguishes between valid and invalid test data and recommends obtaining at least 30 valid tests. In practice, invalid results are often excluded in the analysis, resulting in a loss of information. This paper proposed a BU approach that enables the exploitation of information from “invalid points,” improving accuracy of fracture strength estimations by reducing the bias introduced when excluding “invalid points,” while offering potential enhanced testing efficiency. While the BU approach is general and applicable to any CDR dataset, the quantitative effects observed depend on the dataset characteristics. The following conclusions are drawn:

1. Stronger glass samples have a higher probability of becoming “invalid points,” as they are more likely to fracture outside the inner ring before the true fracture strength within the inner ring region is reached. Consequently, excluding “invalid points” introduces a downward bias in the estimated glass fracture strength distribution as “valid points” alone do not capture higher strength realizations. This censoring process can lead to an underestimation of both variability and average value of glass fracture strength.
2. A Bayesian-updating-based method to incorporate information from invalid tests in glass fracture strength characterization is proposed.
3. The proposed method is applied to two existing databases from the literature—the first at ambient temperature and the second at 275 °C. After incorporating “invalid points,” the posterior predictive distribution for glass fracture strength shifted towards larger mean fracture strengths and exhibited greater standard deviations. The 5% quantile, corresponding to the characteristic value of glass fracture strength, decreased (− 8%) at 25 °C but increased (+ 13%) at 275 °C. These results indicate that, while ignoring “invalid points” generally introduce a bias, the effects of this bias are dataset-specific.
4. The proposed approach incorporating invalid tests improved testing efficiency at 275 °C by achieving the same precision level implicitly required by the standard with fewer tests; at 25 °C, it did not enhance efficiency but helped reducing bias without significantly affecting precision. While the primary advantage of the approach is bias reduction, these results suggest that including “invalid points” may, under certain conditions, also lead to more efficient testing campaigns.
5. The reliability of a glass slab under different load ratios was assessed. It was observed that considering the fracture strength distribution incorporating information from “invalid points” yielded lower values of the reliability index. Incorporating “invalid points” is therefore essential to avoid misleading safety margins and ensure a robust basis for reliability assessment.

In the future, as more data becomes available, the approach capabilities and potential benefit (in terms of enhanced efficiency) need to be further investigated. The approach can also be integrated into optimal experimental design frameworks (e.g., for glazing fracture strength—for instance, at elevated temperatures, where glass properties remain insufficiently understood.

## Data Availability

Data will be made available on request.
